# The Mineral Waters and Health Resorts of Europe

**Published:** 1899-06

**Authors:** 


**The Mineral Waters and Health Resorts of Europe.** By HERMANN WEBER, M.D., and F. PARKES WEBER, M.D. Pp. xiii., 524. London: Smith, Elder, & Co. 1898.

Known in its original issue as *The Spas and Mineral Waters of Europe*, this classical work has undergone much alteration and addition. The two entirely new chapters are amongst the most useful, inasmuch as they deal with the popular inland climatic health resorts, and the various special methods of treatment and sanatoria for phthisis. These resorts are classified in accordance with their elevation above sea level: those of high elevation being from 3500 feet and upwards, the medium class above 1500 and below 3500 feet, and those of slight elevation comprising places below 1500 feet. The localities of high elevation are described as "eminently stimulating and exhilarating and tonic. They promote the expansion of the chest and lungs, and the ventilation of the latter; they improve the appetite, the digestion, the nutrition and the oxygenation and quality of the blood. Under the diminished atmospheric pressure of high altitudes the number of red corpuscles in the blood rapidly increases, as also the percentage of hæmoglobin, though the latter does so more slowly." Accumulating evidence tends to show that we should not be content with any but the localities of high elevation if we wish to give a phthisical patient the chance of a radical cure, and if he is well enough and financially able to attempt it. After describing the dietetic cures and the various sanatoria for special treatment, the author adds the remark that "more sanatoria are undoubtedly needed, both institutions for the treatment of incipient and hopeful cases, and infirmaries for very advanced and very unfavourable cases, where patients who have no private means can be maintained, if necessary for the rest of their lives." Climatic treatment is usually expensive, as is the treatment of phthisis generally; and those who most need it are often those who can the least afford such luxuries as are to them necessities for the maintenance of life.

We are glad to observe that the authors do not encourage the practice of sending severe and advanced cases of diabetes to spas for treatment; they remark that "no complete and permanent cure of established diabetes is known to us from spa treatment"; and, further, "if the term 'diabetes' be accepted in this [Lauder Brunton's] sense, then one must say that only cases of 'glycosuria' are suited to spa treatment." We hold strongly to the belief that the ordinary glycosuria of middle life is most amenable to spa treatment; but that the advanced diabetic should remain at home, and carry on there such dietetic and other treatment as may be possible.

The volume is one which may safely be consulted with the expectation of finding in it sound advice grounded on good judgment and experience.

